# A Rapid Screening Program for Histoplasmosis, Tuberculosis, and Cryptococcosis Reduces Mortality in HIV Patients from Guatemala

**DOI:** 10.3390/jof7040268

**Published:** 2021-04-01

**Authors:** Narda Medina, Ana Alastruey-Izquierdo, Oscar Bonilla, Osmar Gamboa, Danicela Mercado, Juan C. Pérez, Luis Roberto Salazar, Eduardo Arathoon, David W. Denning, Juan Luis Rodriguez-Tudela

**Affiliations:** 1 Mycology Reference Laboratory, National Centre for Microbiology, Instituto de Salud Carlos III, 28220 Madrid, Spain; nardagab@gmail.com (N.M.); anaalastruey@isciii.es (A.A.-I.); 2 Asociación de Salud Integral, 01001 Guatemala City, Guatemala; osmar.gamboa@asigt.org (O.G.); luisro1992@gmail.com (L.R.S.); earathoon@hotmail.com (E.A.); 3 Clínica Familiar “Luis Ángel García”/Hospital General San Juan de Dios, 01001 Guatemala City, Guatemala; oscar.bonilla@asigt.org (O.B.); diagnostico@asi.org.gt (D.M.); jcpsmh@gmail.com (J.C.P.); 4 The National Aspergillosis Centre, University Hospital of South Manchester, Manchester M23 9LT, UK; ddenning@manchester.ac.uk; 5 The University of Manchester and Manchester Academic Health Science Centre, Manchester M23 9LT, UK; 6 Global Action Fund for Fungal Infections, 1208 Geneva, Switzerland

**Keywords:** laboratory diagnosis, opportunistic infections, tuberculosis, histoplasmosis, cryptococcosis

## Abstract

Opportunistic infections (OIs) and advanced HIV disease (AHD) contribute to HIV-related mortality. Here, we analyzed the situation of AHD and OIs in a cohort of newly diagnosed HIV patients from Guatemala. We included 2127 adult patients from 13 facilities across the country during 2017 to 2018. Patients were screened for tuberculosis (TB), nontuberculous mycobacteria (NTM), histoplasmosis, and cryptococcal disease, independently of their CD4 cell count. Of the 2127 enrolled patients, 1682 (79.1%) had a CD4 cell count available; of which 52% presented with AHD. Of the Mayan population, 65% had AHD. The overall OI incidence was 21%. Histoplasmosis was the most frequent OI (7.9%), followed by TB (7.1%); 94.4% of these infections occurred in patients with a CD4 < 350 cells/mm^3^. Mortality at 180 days was significantly higher in those with OIs than without OIs (29.7% vs. 5.9%, *p* < 0.0001). In one year, this program decreased the OI mortality by 7% and increased the OI treatment by 5.1%. Early OI diagnosis and appropriate therapy reduced OI mortality among newly diagnosed HIV patients in Guatemala. Screening for OIs should be considered in all newly diagnosed HIV patients who have a CD4 cell count < 350 cells/mm^3^ or those without a CD4 cell count available. To improve results, interventions such as early HIV detection and access to flucytosine and liposomal amphotericin B are required.

## 1. Introduction

Opportunistic infections (OIs) remain a major cause of death in HIV-infected patients, especially in low- and middle-income countries (LMICs) [1]. Despite the increasing coverage of antiretroviral therapy (ART), 770,000 people died in 2018 [2]. In Latin America (LATAM), these deaths have decreased by 14% since 2010; however, other regions such as eastern and southern Africa have shown a 44% reduction [2]. National and regional data on specific causes of HIV mortality are crucial to develop strategies and to allocate resources effectively.

In 2017, the World Health Organization (WHO) published the first guidelines for managing advanced HIV disease and rapid initiation of antiretroviral therapy [3]. These guidelines recommended a package of interventions oriented to reduce AIDS-related mortality while advising rapid diagnosis and treatment of major OIs. Although these recommendations are focused on people with advanced HIV disease (AHD), there may be benefits of screening strategies that reach all patients at higher risk, such as those newly diagnosed with HIV. Since 2010, the number of new HIV infections has increased by 7% in LATAM [2]. Differences in the burden of OIs have also been described, especially for histoplasmosis, which is an important OI in the region [4,5]. Therefore, we developed a prospective screening study to assess the burden of AHD, the incidence of four OIs, and the mortality at 180 days in a large cohort of newly diagnosed HIV patients in Guatemala.

## 2. Materials and Methods

### 2.1. Study Design and Participants

Patients were enrolled in the OIs program, which was established in Guatemala in 2017. This program encompasses a network of 13 health care facilities (HCFs) and a central Diagnostic Laboratory Hub (DLH) [6]. This work analyzes all newly diagnosed HIV patients enrolled in this program between January 2017 to December 2018 who were over 13 years of age.

### 2.2. Procedures and Definitions

Demographic data were collected in the HCFs using a standard electronic form. Patients were screened for the following OIs: tuberculosis (TB), nontuberculous mycobacteria (NTM), histoplasmosis, and cryptococcosis. Screening was performed independently of the CD4 cell count. The following samples were requested: whole blood in an isolator tube (Abbott Diagnostics, Illinois, Chicago, USA), serum, urine, and sputum. Additional samples were also received based on clinical criteria. Specimens were referred to the DLH to be processed as previously described [6,7]. Laboratory assays included: (i) Smear microscopy; (ii) Sputum culture; (iii) Isolator blood culture; (iv) in-house PCR for *M. tuberculosis* and *H. capsulatum*; (v) Detection of urine antigen of *H. capsulatum* by IgG monoclonal antibodies (Immuno-Mycologics (IMMY), Norman, Oklahoma, USA); and (vi) the cryptococcal antigen lateral flow assay (CrAg) (LFA, IMMY, Norman, Oklahoma, USA). If serum CrAg was positive, a lumbar puncture was advised. The relative diagnostic performance of these tests has been described by Medina et al. [7]. A full screening was considered when the four OIs were analyzed. OI diagnosis required a positive result of at least one of the following techniques: antigen detection, culture, and/or PCR test.

Disseminated histoplasmosis was considered when an Isolator blood culture and/or urine antigen was positive, and cryptococcal meningitis when we detected a positive CrAg test or isolated *C. neoformans* in cerebrospinal fluid (CSF). AHD was defined as having a CD4 count < 200 cells/mm^3^. CD4 cell counts were obtained by the different HCFs. An available CD4 cell count was defined as a cell count obtained 90 days before or after the moment of the OI screening. Patients from Guatemala City and other cities were categorized as urban while other areas were defined as rural.

Patients were treated in the HCFs and antiretroviral therapy was started in accordance with the national guidelines [8,9].

### 2.3. Ethics Statement

Because this study analyzed data obtained during the implementation of a routine program that provided diagnostic services to newly diagnosed HIV patients, informed consent was not requested for the OI screening; however, a written informed consent for HIV testing was obtained by the HCFs of the network. For the analysis, no personally identifiable data information was collected, and confidentiality of patients was assured.

### 2.4. Data Analysis

The participants were categorized according to the OI diagnosis. Baseline characteristics were compared with chi-square or Fisher’s exact test for categorical variables and Mann-Whitney U-test for continuous variables. We performed univariate and multivariable analyses to identify demographic factors associated with AHD. Receiver operator curves (ROCs) were carried out to evaluate different CD4 thresholds among the OI cases for screening purposes. Sensitivity, specificity, and their two-sided 95% confidence intervals (95% CI) were determined.

## 3. Results

### 3.1. Study Population

A total of 2127 newly diagnosed HIV patients were enrolled between January 2017 and December 2018. This study encompassed 58.3% (2127 out of 3646) of the new HIV infections reported by the HIV national program. Table 1 summarizes the patients’ characteristics. The median age at HIV diagnosis was 31 years (IQR: 25–41) and 70.3% of the patients were male. CD4 cell counts were available for 1682 patients (79.1%), of those, 877 (52.1%) had AHD. Women and men had similar CD4 cell counts (median, 244 vs. 228; *p* = 0.623); however, heterosexual men had significantly lower CD4 counts than men who have sex with men (MSM) (median CD4, 124 cells/mm^3^ vs. 264 cells/mm^3^; *p* < 0.0001). The difference of AHD between the Mayan and Ladino population was statistically significant (65.8% vs. 49.7%, *p* < 0.0001), but no difference was observed in OI rate (18% vs. 17.5%, *p* = 0.282). Mayan women and men had similar CD4 cell counts (median CD4, 160 cells/mm^3^ vs. 125 cells/mm^3^; *p* = 0.411).

Among 877 patients with AHD, 322 (36.7%) had CD4 cell counts between 0–49 cells/mm^3^, 202 (23%) between 50–99 cells/mm^3^, and 353 (40.2%) between 100–199 cells/mm^3^. In the univariate analysis, risk factors associated with AHD were older age at the moment of diagnosis, belonging to a Mayan ethnic group, heterosexual category, and patients who live in rural areas. In the multivariate analysis, the risk factors significantly associated with AHD were: (i) age 30–50 years (aOR 1.5, 95% CI (1.2–1.9), *p* < 0.0001), (ii) >50 years (aOR 2.3, 95% CI (1.6–1.9), *p* < 0.0001), and (iii) Mayan ethnic group (aOR 1.5, 95% CI (1.1–2.0), *p* = 0.009). Compared with heterosexuals, MSM were less associated with AHD (aOR 0.48, 95% CI (0.37–0.62), *p* < 0.0001).

### 3.2. Opportunistic Infections

Of the 2127 participants, 1821 (85.6%) had full screening done. Three hundred eighty-five patients (18.1%) had OIs: 140 (36.4%) histoplasmosis, 121 (31.4%) TB, 78 (20.3%) cryptococcal disease, 31 (8.1%) multiple OIs, and 15 (3.9%) NTM infection. In patients with multiple OIs, we found histoplasmosis/cryptococcosis (35.5%), histoplasmosis/tuberculosis (32.3%), and cryptococcosis/tuberculosis (12.9%) as the most frequent. In 2018, the incidence of OIs increased 3.9% (from 15.9% to 19.8%). Patients with OIs were significantly older (median, 35 years vs. 30 years; *p* < 0.0001), tended to be heterosexual (79.5% vs. 63.9%; *p* < 0.0001), and had higher HIV viral load (Log_10_ 5.2 OIs vs. 4.7 *p* < 0.0001) than those without OIs (Table 1). OIs were more frequent in patients living in rural areas than those living in urban zones (10% vs. 5.8%, *p* = 0.018). Among country regions, histoplasmosis and cryptococcal disease were frequent in the East (10.1% and 8.1%), West (10.9% and 5.7%), and Central regions (8.8% and 4.5%). In Guatemala City, TB was more frequent (8.3%).

As expected, the incidence of an OI was inversely correlated with CD4 cell count (Table 2). Histoplasmosis was the most frequent AIDS-defining illness. The difference between histoplasmosis and TB incidence was statistically significant in patients with CD4 cell counts <50 cells/mm^3^ (19.7% vs. 11.5%, *p* = 0.003). Among those with AHD, the incidence of OIs was 30.3%. In patients who had a CD4 cell count ≥ 350 cells/mm^3^, TB was more frequent than histoplasmosis (2.9% vs. 1.1%, *p* = 0.0807) (Table 2).

We analyzed, by means of a ROC curve, OIs (Yes/No) against CD4 cell counts to determine the best threshold to screen patients for OIs. The AUC was 0.771 (95%CI; 0.741–0.801). Table 3 shows the sensitivity and specificity of the different thresholds. Almost 95% of the OIs investigated occurred in patients with <350 CD4/mm^3^. Fifteen OIs were diagnosed in patients with ≥350 CD4 cells/mm3: 10 TB, 4 histoplasmosis, and 1 cryptococcal disease. Eighty-one (63.3%) of 128 patients with disseminated histoplasmosis had a CD4 cell count available. If histoplasmosis screening had been limited to patients with CD4 cell counts < 100 or <200 CD4 cells/mm^3^, 16 (19.7%) and 6 (7.4%) cases would have been missed, respectively. For cryptococcal disease, 5 (13.8%) and 1 (2.8%) cases would have been missed, respectively. A total of 117 (30.4%) OIs were diagnosed in patients without a CD4 cell count result available.

### 3.3. Treatment and Outcome

A total of 324 (84.1%) patients with OIs were treated. Information was available for 36 (59%) out of the 61 patients who did not receive treatment: 18 (50%) died before initiation of treatment, 12 (33.3%) were lost to follow-up, and 6 (16.7%) refused treatment (median CD4, 88 cells/mm^3^). Treatment information for the OI cases is shown in Table 4.

After 180 days of enrollment, 213 (10%) patients died. The OIs investigated in this program accounted for 111 of the 213 deaths (52.1%): 18.3% were due to histoplasmosis, 12.7% to TB, 11.3% to cryptococcosis, 7.0% to multiple OIs, and 2.8% to NTM. Unknown etiology accounted for 24.9% of deaths and other causes for 23.0%. One hundred fifteen deaths (53.9%) occurred in the first month after enrollment.

**Figure 1:**
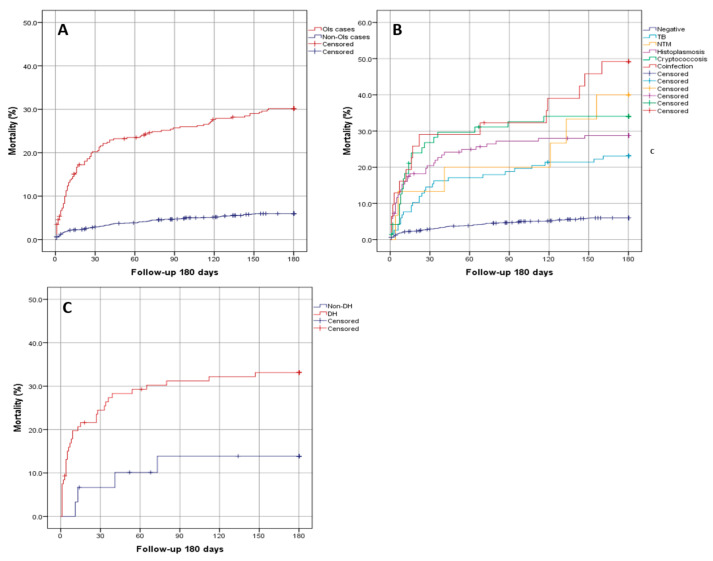
Kaplan–Meier survival curves. (**A**) Patients with and without opportunistic infections. (**B**) Patients without opportunistic infections, with tuberculosis, nontuberculous mycobacteria, cryptococcal disease, histoplasmosis, and with multiple opportunistic infections. (**C**) Patients with disseminated histoplasmosis and non-disseminated histoplasmosis.

Figure 1A shows the significantly higher mortality in patients with OIs than those without OIs (29.7% vs. 5.9%; *p* < 0.0001). In 2018, mortality from OIs was 7% lower than in 2017 (34% vs. 27%; *p* = 0.187). Regarding each OI, and including cases of multiple infections, patients with tuberculosis had a lower mortality in 2018 compared to 2017 (18.5% vs. 34.6%, *p* = 0.039) with a similar trend for cryptococcal disease (32.8% vs. 46.9%, *p =* 0.210); treatments for these infections increased in 2018 compared to 2017 (TB, 87.3% to 97.6% (*p* = 0.022); and cryptococcal disease 67.6% to 90.2% (*p* = 0.008)). In NTM cases, mortality increased from 17.5% in 2017 to 42.5% in 2018 (*p* = 0.126), with fewer patients receiving treatment (80% vs. 37.5%); 4 deaths (60%) occurred between 90–180 days. In patients with multiple OIs, overall mortality decreased 16.2% (from 58.3% to 42.1%, *p =* 0.469). Histoplasmosis mortality was similar in 2017 and 2018 (32.8% and 32%, *p* = 0.965), but 3% fewer patients were treated in 2018 (84.6% to 81.6%, *p* = 0.392). In patients without OIs, mortality rate was lower in MSM compared with heterosexuals (2% vs. 6.9%, *p* < 0.0001). Figure 1B shows the cumulative probability of death by each OI at 180 days. A mortality rate of 48.4% was observed in patients with multiple OIs followed by NTM (40%), cryptococcal disease (32.9%), histoplasmosis (28.3%), and TB (23.1%) (*p* < 0.0001). Of those with multiple OIs and a CD4 cell count available, 24 (95.8%) had AHD. Concerning histoplasmosis (Figure 1C), the mortality rate was higher in those with disseminated histoplasmosis than non-disseminated cases (32.7% vs. 13.3%, *p* < 0.0001). Mortality was also higher in patients with cryptococcal meningitis than those with a negative lumbar puncture (34.4% vs. 8.3%; *p* = 0.141). However, in patients with a positive CrAg in serum who did not undergo a lumbar puncture, the mortality was 37% (10 out of 27 cases).

## 4. Discussion

This study analyzes the situation of newly diagnosed HIV patients in Guatemala. The data are able to provide robust evidence since they account for 58.3% (2127 out of 3646) of the new HIV infections reported by the national HIV program during 2017–2018. The proportion of the newly diagnosed HIV patients presenting with AHD was 52%, which was 6% higher than the estimated frequency by UNAIDS of 46% [2]. In other LATAM countries, AHD ranged from 20 to 40% [2]. Several studies in Asia (36.3%), Ethiopia (39%), South Africa (35.6%), and Rwanda (29.4%) also showed lower rates of AHD [10,11,12,13,14]. Therefore, Guatemala has one of the highest rates of newly diagnosed HIV patients presenting with AHD in the world. This rate was especially high in the Mayan population with 65.8%. A previous study in Guatemala found lower levels of HIV knowledge in the Mayan population compared with Ladinos [15]. Thus, limited information and structural disadvantages could explain the late diagnosis. We also found that heterosexual patients were more likely to be associated with AHD at the moment of diagnosis compared with MSM, similar to results found in other studies [16,17]. This is possibly due to awareness campaigns and active HIV screening programs implemented in recent years that have promoted an earlier diagnosis in MSM. Overall, the high frequency of AHD underscores the urgent need for national campaigns and adapted HIV testing strategies.

To diagnose OIs in newly diagnosed HIV patients, the WHO recommends screening for cryptococcal antigen in patients with CD4 cell counts < 100 cells/mm^3^ and routine clinical evaluation for TB [3,18]; however, we screened all patients irrespective of their CD4 cell count. Our results showed that 27.9% of OIs occurred in patients with CD4 cell counts > 100 cells/mm^3^ (Table 2). The ROC analysis using CD4 cell counts <200 cells/mm^3^ as the screening threshold showed a sensitivity of 85.1% and in patients with <350 cells/mm^3^ a sensitivity of 94.4%. The number needed to test is six patients to diagnose one case of OI at CD4 count of 100–200 cells/mm^3^ and 15 at a CD4 count of 200–350 cells/mm^3^. For this reason, we cannot recommend a CD4 cell count <200 cells/mm^3^ as the screening threshold since almost 15% of the OIs would have been missed. Considering that OIs are usually life-threatening diseases, we find it reasonable to spend $7.5 on cryptococcal antigen tests and $13 on histoplasmosis testing. Furthermore, TB should be investigated independently of the CD4 cell count.

Histoplasmosis was the most common OI with an overall incidence of 7.9% rising to 19.7% in patients with CD4 cell counts < 50 cells/mm^3^. Guatemala is known to be a hyperendemic area for histoplasmosis [4,5,19]. Here, we found almost double the incidence than the estimated in a previous study of the histoplasmosis burden in LATAM (4.1% vs. 7.9%) [4]. In June 2020, the Pan American Health Organization (PAHO) published guidelines for diagnosing and managing disseminated histoplasmosis in people living with HIV [20]. This document recommends urine antigen detection as the reference technique to diagnose disseminated histoplasmosis [20]. In Guatemala, a comparative study of the laboratory assays showed the highest rate of detection for histoplasmosis with the urine antigen test [7]. In addition, it is clinically difficult to determine whether the patient has histoplasmosis or tuberculosis. Therefore, the screening approach provides the means to differentiate both OIs. Concerning cryptococcal disease, current estimations in LATAM are limited. A previous study of the global burden of HIV-associated cryptococcal meningitis estimated the overall prevalence of cryptococcal antigen in patients with CD4 cell counts < 100 cells/mm^3^ to be 6% [21]. Here, we found that 8.2% of AHD patients had a positive serum CrAg test. Hence, it seems that the burden of histoplasmosis and cryptococcal disease have been underestimated in the region. For TB cases, a previous report by the Pan American Health Organization (PAHO) estimated an incidence of 9.9% among HIV patients in Guatemala in 2014 [22] This was 1.95% higher than the overall TB incidence found in this study (9.9% vs. 7.1%). In our cohort, the incidence of TB was 0.8% lower than histoplasmosis. Considering that TB is more frequent than histoplasmosis in urban areas and one of the largest HCFs, which is located in Guatemala City, did not participated in this study, we can hypothesize that the incidence of TB might be higher.

Mortality among newly diagnosed HIV patients was high with 52.1% of deaths attributed to the OIs screened. A substantial number of patients also died with an unknown diagnosis. This should be improved with the expansion of the diagnostic portfolio. It is important to highlight that the diagnosis has to be made quickly since 53.9% of deaths occurred in the first 30 days. The highest mortality rate was observed in patients with multiple OIs (48.4%). Contributing factors to this increased mortality could include severe immunosuppression (96% had AHD), complicated management, and drug–drug interactions [23]. Histoplasmosis was also associated with a high mortality, similar to previous analyses (28.3%) [24,25]. We found a 7% reduction in the OI mortality in 2018 compared to 2017, with a simultaneous increase in the OI treatment (5.1%). This finding should be attributed to the early diagnostic capacity provided by the OI program. Nevertheless, mortality of cryptococcal meningitis was 34%. It is well known that amphotericin B plus flucytosine decreases cryptococcal meningitis mortality [26,27], yet flucytosine is not available in Guatemala. Therefore, its introduction into the country should be a priority. In addition, the mortality rate in patients with cryptococcal antigenemia without a lumbar puncture (LP) was similar to those with meningitis (37% vs. 34%), suggesting that better management of these patients should be instituted. Reasons for not realizing LP in these patients was not recorded. Since the introduction of the *Histoplasma* urine antigen test, the associated mortality has decreased by 8.3% (From 36.6% in a reference HCF to 28.3% in the network) [28]. However, liposomal amphotericin B is not available in Guatemala, and mortality could be further decreased with its introduction. Concerning NTM, 15 cases were diagnosed with a high mortality rate (40%). Here, NTM identification requires the growth of the microorganism, which can take several weeks. Thus, rapid methods such as GenoType CMdirect (Hain Lifescience) that can be performed from direct specimens would be required.

The present study has its limitations. The OI status was based on the available laboratory techniques in this program, but other OIs have been missed. This will have led to an underestimation of the burden of OIs. Other factors such as ARV treatment were not determined. The integration of all HCFs in the network will improve the accuracy of the data. Despite the limitations, our findings show that Guatemala has one of the highest rates of AHD in newly diagnosed HIV patients, the burden of OIs among newly diagnosed HIV patients is substantial, the burden of fungal infections has been previously underestimated, and access to rapid diagnosis of OIs has decreased the overall mortality by 7% in a single year, showing that it is an essential tool to achieve the goal of eliminating AIDS and reducing deaths.

## Figures and Tables

**Table 1:** Baseline characteristic of newly diagnosed HIV patients.

Characteristic	Total 2127 (100%)		With OI 385 (18%)		Without OI 1742 (82%)	
	*n*	%	*n*	%	*n*	%
Sex						
Male	1495	70.3	280	72.7	1215	69.7
Female	608	28.6	104	27.0	504	28.9
Transsexual	24	1.1	1	0.3	23	1.3
Age (years)						
*n*	2121	99.7	380	98.7	1741	99.9
Median, IQR	31	(25–41)	35	(28–45)	30	(24–40)
Sexual orientation						
Heterosexual	1420	66.8	306	79.5	1114	63.9
Homosexual	486	22.8	39	10.1	447	25.7
Bisexual	163	7.7	19	4.9	144	8.3
Unknown	58	2.7	21	5.5	37	2.1
Ethnic group						
Ladino	1524	71.7	266	69.1	1258	72.2
Mayan	334	15.7	60	15.6	274	15.7
Other	13	0.6	4	1.0	9	0.5
Unknown	256	12.0	55	14.3	201	11.5
Residence	2077	97.6	371	96.3	1706	97.9
Urban	995	47.9	159	42.9	836	49
Rural	1082	52.1	212	57.1	870	51
BMI						
*n*	1198	56.3	185	48.0	1013	58.1
Underweight	148	12.4	45	24.5	103	10.2
Normal	695	58.1	98	53.3	597	58.9
Overweight	275	23.0	33	17.9	242	23.9
Obese	79	6.6	8	4.3	71	7.0
CD4 (cells/mm^3^)						
*n*	1682	79.1	268	69.6	1414	81.2
Median, IQR	187	(73–326)	53	(19–137)	220	(102–359)
<200	877	52.1	227	84.7	650	46.0
<350	1298	77.2	253	94.4	1045	73.9
≥350	384	22.8	15	5.6	369	26.1
Viral load (copies/mL)						
*n*	1668	78.4	277	72.0	1391	79.8
Log_10_ Median, IQR	4.8	(4.2–5.3)	5.2	(4.7–5.7)	4.7	(4.1–5.2)

**Table 2:** Opportunistic infections incidence stratified by CD4 cell count.

OIs	Overall Incidence	Interval CD4 Cell Count				
		<50	50–99	100–199	200–350	>350
Tuberculosis	7.1%	11.5%	10.8%	7.5%	2.7%	2.9%
NTM	1.1%	2.4%	0.5%	1.9%	0.3%	0.0%
Histoplasmosis *	7.9%	19.7%	7.0%	4.1%	2.4%	1.1%
Cryptococcosis ^+^	4.8%	14.3%	7.4%	3.3%	1.5%	0.3%
Total	21.0%	47.9%	25.8%	16.8%	6.8%	4.3%

* All cases of histoplasmosis (disseminated and non-disseminated) were included; ^+^ All cryptococcal cases (meningitis and non-meningitis) were included.

**Table 3:** CD4 cell counts thresholds. Sensitivity and specificity for OIs screening.

CD4 Threshold	Sensitivity (95% CI)	Specificity (95% CI)
<100	66.6 (59.8–71.1)	75.2 (73.1–77.6)
<200	85.1 (80–88.5)	53.9 (51.4–56.6)
<350	94.4 (90.1–96.6)	26.0 (23.8–28.4)

**Table 4:** Opportunistic infection treatments.

Treatments	Total	TB	NTM	Histo	Crypto	Coinfections
	(*n* = 385)	(*n* = 121)	(*n* = 15)	(*n* = 140)	(*n* = 78)	(*n* = 31)
	*n* (%)	*n* (%)	*n* (%)	*n* (%)	*n* (%)	*n* (%)
Antifungal therapy	206 (53.5)	-	-	116 (82.9)	64 (82)	25 (80.6)
Amphotericin B	141 (36.6)	-	-	89 (63.6)	39 (50)	13 (41.9)
Itraconazole	74 (19.2)	-	-	67 (47.9)	2 (2.5)	5 (16.1)
Fluconazole	75 (19.5)	-	-	7 (5)	52 (66.7)	12 (38.7)
Anti-tuberculous drugs	127 (33)	112 (92.5)	-	-	-	12 (38.7)
Antibiotics	11 (2.9)	-	2 (13.3)	-	-	2 (6.4)

Abbreviations: Crypto, cryptococcal disease; Histo, histoplasmosis; NTM, nontuberculous mycobacteria; TB, tuberculosis.

## Data Availability

The data presented in this study are available on request from the corresponding author.

## References

[1] Low A., Gavriilidis G., Larke N., B-Lajoie M.-R., Drouin O., Stover J., Muhe L., Easterbrook P. (2016). Incidence of Opportunistic Infections and the Impact of Antiretroviral Therapy Among HIV-Infected Adults in Low-and Middle-Income Countries: A Systematic Review and Meta-analysis. Clin. Infect. Dis..

[2] Unaids (2019). UNAIDS Data 2019.

[3] World Health Organization (2017). Guidelines for Managing Advanced HIV Disease and Rapid Initiation of Antiretroviral Theraphy.

[4] Adenis A.A., Valdes A., Cropet C., Mccotter O.Z., Derado G., Couppie P., Chiller T., Nacher M. (2018). Burden of HIV-associated histoplasmosis compared with tuberculosis in Latin America: A modelling study. Lancet Infect. Dis..

[5] Medina N., Samayoa B., Lau-Bonilla D., Denning D.W., Herrera R., Mercado D., Guzmán B., Pérez J.C., Arathoon E. (2017). Burden of serious fungal infections in Guatemala. Eur. J. Clin. Microbiol. Infect. Dis..

[6] Samayoa B., Aguirre L., Bonilla O., Medina N., Lau-Bonilla D., Mercado D., Moller A., Perez J.C., Alastruey-Izquierdo A., Arathoon E. (2020). The Diagnostic Laboratory Hub: A New Health Care System Reveals the Incidence and Mortality of Tuberculosis, Histoplasmosis, and Cryptococcosis of PWH in Guatemala. Open Forum Infect. Dis..

[7] Medina N., Alastruey-izquierdo A., Mercado D., Aguirre L., Samayoa B., Bonilla O., Pérez J.C., Rodriguez-tudela J.L. (2020). Comparative performance of the laboratory assays used by a Diagnostic Laboratory Hub for opportunistic infections in people living with HIV. AIDS.

[8] Ministerio de Salud Pública y Asistencia Social de Guatemala (2019). Guía de uso de los antirretrovirales en personas con VIH y su aplicación profiláctica.

[9] Ministerio de Salud Pública y Asistencia Social de Guatemala (2018). Manual de atención para el manejo del paciente con Tuberculosis.

[10] Kiertiburanakul S., Boettiger D., Lee M.P., Fs Omar S., Tanuma J., Ng O.T., Durier N., Phanuphak P., Ditangco R., Chaiwarith R. (2014). Trends of CD4 cell count levels at the initiation of antiretroviral therapy over time and factors associated with late initiation of antiretroviral therapy among Asian HIV-positive patients. J. Int. AIDS Soc..

[11] Melaku Z., Lamb M.R., Wang C., Lulseged S., Gadisa T., Ahmed S., Habtamu Z., Alemu H., Assefa T., Abrams E.J. (2015). Characteristics and outcomes of adult Ethiopian patients enrolled in HIV care and treatment: A multi-clinic observational study. BMC Public Health.

[12] Burden H., Carmona S., Bor J., Nattey C., Maughan-Brown B., Maskew M., Fox M.P., Glencross D.K., Ford N., Macleod W.B. (2018). High Burden of Advanced HIV Disease Among Patients Seeking Care in South Africa’s National HIV Program: Data From a Nationwide Laboratory Cohort. Clin. Infect. Dis..

[13] Mutimura E., Addison D., Anastos K., Hoover D., Dusingize J.C., Karenzie B., Izimukwiye I., Mutesa L., Nsanzimana S., Nashi D. (2015). Trends in and correlates of CD4 + cell count at antiretroviral therapy initiation after changes in national ART guidelines in Rwanda. AIDS.

[14] Lahuerta M., Wu Y., Hoffman S., Elul B., Kulkarni S.G., Remien R.H., Nuwagaba-Biribonwoha H., El-Sadr W., Nash D. (2014). Findings From Four Sub-Saharan African Countries for the Multi-level determinants of late ART initiation in sub-Saharan Africa Team and the Identifying Optimal Models of HIV Care in sub-Saharan Africa Collaboration. Clin. Infect. Dis..

[15] Taylor T.M., Hembling J., Bertrand J.T. (2015). Ethnicity and HIV risk behaviour, testing and knowledge in Guatemala. Ethn. Health.

[16] Hu X., Liang B., Zhou C., Jiang J., Huang J., Ning C., Liu J., Zhou B., Zang N., Lai J. (2019). HIV late presentation and advanced HIV disease among patients with newly diagnosed HIV/AIDS in Southwestern China: A large-scale cross-sectional study AIDS Research and Therapy. AIDS Res. Ther..

[17] Meléndez J., Reinhardt S.W., O’Halloran J.A., Spec A., Alonzo Cordon A., Powderly W.G., Mejia Villatoro C. (2019). Late Presentation and Missed Opportunities for HIV Diagnosis in Guatemala. AIDS Behav..

[18] World Health Organization (2018). Guidelines for the Diagnosis, Prevention and Management of Cryptococcal Disease in HIV-Infected Adults, Adolescents and Children.

[19] Taylor R.L., Dobrovolny C.G. (1960). The Distribution of Histoplasmin Sensitivity in Guatemala. Am. J. Trop. Med. Hyg..

[20] Pan American Health Organization (2020). Diagnosing and Managing Disseminated Histoplasmosis among People Living with HIV.

[21] Rajasingham R., Smith R.M., Park B.J., Jarvis J.N., Govender N.P., Chiller T.M., Denning D.W., Loyse A., Boulware D.R. (2017). Global burden of disease of HIV-associated cryptococcal meningitis: An updated analysis. Lancet Infect. Dis..

[22] Pan American Health Organization (2014). Tuberculosis in the Americas.

[23] Agudelo C.A., Restrepo C.A., Molina D.A., Tobón A.M., Kauffman C.A., Murillo C., Restrepo A. (2012). Tuberculosis and histoplasmosis co-infection in AIDS patients. Am. J. Trop. Med. Hyg..

[24] Adenis A., Nacher M., Hanf M., Vantilcke V., Boukhari R., Blachet D., Demar M., Aznar C., Carme B., Couppie P. (2014). HIV-Associated Histoplasmosis Early Mortality and Incidence Trends: From Neglect to Priority. PLoS Negl. Trop. Dis..

[25] Falci D.R., Monteiro A.A., Braz Caurio C.F., Magalhães T.C.O., Xavier M.O., Basso R.P., Melo M., Schwarzbold A.V., Ferreira P.R.A., Vidal J.E. (2019). Histoplasmosis, An Underdiagnosed Disease Affecting People Living With HIV/AIDS in Brazil: Results of a Multicenter Prospective Cohort Study Using Both Classical Mycology Tests and Histoplasma Urine Antigen Detection. Open Forum Infect. Dis..

[26] Loyse A., Thangaraj H., Easterbrook P., Ford N., Roy M., Chiller T., Govender N., Harrison T.S., Bicanic T. (2013). Cryptococcal meningitis: Improving access to essential antifungal medicines in resource-poor countries. Lancet Infect. Dis..

[27] Flucytosine and Cryptococcosis: Time to Urgently Address the Worldwide Accessibility of a 50-Year-Old Antifungal. https://www.ncbi.nlm.nih.gov/pmc/articles/PMC3797641/.

[28] Samayoa B., Mercado D., Scheel C., Guazmán B., Amado I., Gómez B., Morales R.E., Chiller T., Arathoon E., Recinos A. (2012). Disseminated histoplasmosis (DH) before and after the implementation of urine antigen detection ELISA (UADE) in an HIV clinic in Guatemala. ICAAC.

